# Bearing Witness: *Témoignage* as a Tool for Child Advocacy during Armed Conflict

**DOI:** 10.1371/journal.pgph.0004947

**Published:** 2025-09-10

**Authors:** Anik Patel, Amber Alayyan, Heather A Haq, Parminder S Suchdev, Naji Hattar, Jeffrey Goldhagen, David J Schonfeld, Tanya Haj-Hassan, Lia Harris, Seema Jilani, Ayesha Kadir, Paul H. Wise, Lisa Umphrey

**Affiliations:** 1 Department of Pediatrics, Children’s Mercy Kansas City, Kansas City, Missouri, United States of America; 2 Médecins sans Frontières, Operational Center Paris, Paris, France; 3 Department of Pediatrics, Baylor College of Medicine, Houston, Texas, United States of America; 4 Department of Pediatrics, Emory University, Atlanta, GeorgiaUnited States of America; 5 Independent Researcher, Chicago, Illinois, United States of America; 6 Department of Pediatrics, University of Florida College of Medicine, Jacksonville, Florida, United States of America; 7 National Center for School Crisis and Bereavement, Children’s Hospital Los Angeles, Los Angeles, California, United States of America; 8 Médecins sans Frontières, Operational Center Geneva, Geneva, Switzerland; 9 Department of Pediatrics, University of British Columbia, Vancouver, British Columbia, Canada; 10 Viborg Regional Hospital, Denmark; 11 Department of Pediatrics, Stanford School of Medicine, Stanford, California, United States of America; 12 Department of Pediatrics, University of Colorado, Aurora, Colorado, United States of America; World Health Organization, ETHIOPIA

## Abstract

Children affected by armed conflict suffer devastating physical, emotional, and social harm. War uproots families, forcing many to flee as refugees or internally displaced persons, while others remain trapped in dangerous environments. In these crises, children face disproportionate risks—violence, exploitation, disrupted education, and collapsed healthcare systems. Their unique vulnerabilities require urgent, targeted action to protect their health, rights, and development. Beyond immediate care, the humanitarian principle of témoignage—bearing witness—is essential. Rooted in humanitarian ethics, témoignage means speaking out about injustice, amplifying the voices of those affected, and driving systemic change. It challenges traditional notions of neutrality and calls on humanitarian professionals to ethically advocate for those they serve. Pediatricians and pediatric organizations have a moral duty to ensure that children affected by conflict are seen, heard, and not forgotten. This commentary calls for recognizing children’s distinct humanitarian rights and urges global pediatric societies to take action. To guide this effort, the paper introduces a framework of seven pillars of pediatric témoignage: 1. Amplifying children’s voices, 2. Advocating for systemic justice, 3. Providing trauma-informed care, 4. Supporting education and psychosocial integration, 5. Advancing training and research, 6. Building professional and community networks, and 7. Creating platforms for policy influence. These pillars offer a shared language and practical strategies for pediatricians to document harm, collaborate with advocacy groups, and speak out in public forums. Through témoignage, pediatricians can help protect children’s dignity and rights, ensure their suffering is not normalized, and contribute to a more just and responsive global system for children in conflict.

## Introduction

*“There is no trust more sacred than the one the world holds with children. There is no duty more important than ensuring that their rights are respected, that their welfare is protected, that their lives are free from fear and want and that they can grow up in peace.”* - Kofi Annan, former UN Secretary General [1]

Armed conflict settings are characterized by complex and often protracted cycles of social, economic, and environmental instability, each phase having a profound impact on the lives of children and their communities. [2–4] Conflict settings encompass areas with active wars but also areas affected by terrorism, gang violence, [5] and military occupation, where civilian populations face instability, violence, restricted movement, impeded access to essential services, displacement, immediate and enduring trauma and grief, and heightened vulnerability to human rights violations.

The most vulnerable populations in armed conflict settings are children (which we define as any infant, child, or adolescent younger than 18 years of age). [3] Worldwide, over 473 million children are now living in conflict zones. [6] They are targeted, exploited, injured, maimed, tortured, raped, or killed by combatants. [4] They are recruited and forced to engage in conflicts as combatants themselves. [7] Their schools and playgrounds are reduced to rubble. [8] Their homes are destroyed and their families torn apart by violence, death, or by the impossible choice between fleeing for safety or staying in danger. [2] They are denied access to medical care, vaccinations, and adequate nutrition. [4] Despite the magnitude of their suffering, the plight of children in armed conflict settings is often overlooked, particularly in prolonged or protracted conflict, [4,9,10] and their pain permeates communities, causing intergenerational trauma and grief long after the guns fall silent. [11]

Every child has a right to be heard, but many children need advocates to elevate their voices. Advocates come in many forms, including healthcare providers, community health workers, social workers, lawyers, and teachers. Advocacy can take many forms, from public messaging and media engagement to behind-the-scenes policy negotiations and meetings with government officials. As pediatricians, we are uniquely positioned to amplify children’s voices in our professional interactions with them and their families. For children in conflict settings, speaking out against their suffering becomes even more critical, often serving as a course of action most immediately available to pediatricians. Regardless of individual political beliefs, pediatricians from all backgrounds share a fundamental commitment to ensuring the well-being of children.

This commentary is a call to action for all pediatricians and pediatric societies to unite around our common, shared purpose: the unwavering commitment to uphold and protect children’s distinct, universal, and unique rights to health and well-being. It asserts that armed conflict threatens these rights, which demand urgent recognition to guarantee the special protection of children. It further emphasizes that *témoignage*, the act of bearing witness and speaking out, is a crucial strategy to protect the rights of children impacted by conflict. Our author group represents pediatricians, humanitarians, aid workers, researchers, educators, and activists from varied institutions, backgrounds, and organizations. Our experience with children in conflict settings spans decades and across the globe. While we highlight certain conflicts or contexts, this does not reflect bias or imply the exclusion of others, but rather these examples illustrate broader principles relevant to all conflict settings. Together, we seek to ensure that the rights of children in conflict settings are upheld, demand that their special status be recognized, and propose actionable recommendations for pediatricians related to speaking out and advocacy. Pediatricians bear witness to the impact of armed conflict on children, and we share a professional responsibility and duty to respond and act.

## The special status and claims of children during armed conflict

*“There can be no keener revelation of a society’s soul than the way in which it treats its children.”* – Nelson Mandela, anti-Apartheid activist, former president of South Africa [12]

Children have special status that demands unique protection during armed conflicts. Safeguarding children’s rights and ensuring the enforcement of international laws that protect them not only benefits children but also strengthens the broader humanitarian response for all civilians, contributing to the long-term stability and recovery of affected populations. [13] To effectively ensure children’s special status, it is imperative to ground protective measures in a robust framework of conventions, treaties, and legal concepts relevant to violence against children in conflict. Examples include the Charter of the United Nations (UN) (1945), [14] the Universal Declaration of Human Rights (1948), [15] the Geneva Conventions (1949) and their Additional Protocols (1977), [16] United Nations Convention on the Rights of the Child (UNCRC, 1989), [17] and the Optional Protocol to the CRC on the involvement of children in armed conflict (2000) [18]. In Table 1, we present these works and additional key terms and concepts essential for understanding and discussing children’s rights in armed conflict. Additionally, we present the most egregious violations against children in conflict settings, established through UN Security Council Resolutions and unanimously approved in 2005 and termed the “Six Grave Violations” [9,19] (Table 2); these acts of brutality represent the most atrocious crimes committed against children during armed conflict.

**Table 1:** Definitions and key concepts relevant to children in armed conflict settings.

Abduction [9]	Unlawful removal, seizure, capture, apprehension, taking or enforced disappearance of a child either temporarily or permanently for the purpose of exploitation.
**Armed conflict** [9,20]	Any organized dispute where armed force is used by an organized actor against another organized actor, or against civilians, resulting in at least 25 battle-related deaths in one calendar year. **International armed conflicts (IACs)** occur when one or more States have recourse to armed force against another State, regardless of the reasons or the intensity of this confrontation. **Non-international armed conflicts (NIACs**) are protracted armed confrontations occurring between governmental armed forces and the forces of one or more armed groups, or between such groups arising on the territory of a State party to the Geneva Conventions. The armed confrontation must reach a minimum level of intensity and the parties involved in the conflict must show a minimum level of organization that allows them to plan and carry out coordinated military operations. When both of these criteria are met, then it is legally classified as a non-international armed conflict and international humanitarian law therefore applies.
**Asylum seeker** [20]	A person who seeks safety from persecution or serious harm in a country other than his or her own and awaits a decision on the application for refugee status under relevant international and national instruments.
**Child or Girl Associated with an Armed Force or Armed Group** [7]	Preferred term over “child soldier”; any person below 18 years of age who is, or who has been, recruited or used by an armed force or armed group in any capacity, including but not limited to children, boys and girls, used as fighters, cooks, porters, spies or for sexual purposes.
**Crimes Against Humanity** [21]	Include multiple acts when committed as part of a widespread or systematic attack directed against any civilian population, with knowledge of the attack. Crimes against humanity have not yet been codified in a dedicated treaty of international law, unlike genocide and war crimes.
**Displacement** [2]	Process in which people are compelled to flee or leave their homes or places of habitual residence in order to avoid the effects of armed conflict.
**Ethnic cleansing** [21]	Forced removal of an ethnic group from a territory; defined as “rendering an area ethnically homogeneous by using force or intimidation to remove persons of given groups from the area.” Not recognized as a standalone crime under international law, but the practice may constitute genocide, crimes against humanity, or war crimes.
**Genocide** [21]	Specific acts committed with intent to destroy, in whole or in part, a national, ethnic, racial or religious group, such as: a.Killing members of the group. b.Causing serious bodily or mental harm to members of the group. c.Deliberately inflicting on the group conditions of life calculated to bring about its physical destruction in whole or in part. d.Imposing measures intended to prevent births within the group. e.Forcibly transferring children of the group to another group.
**Internally displaced people** [20]	People who have been forced or obliged to flee or to leave their homes or places of habitual residence, in particular as a result of or to avoid the effects of armed conflict, situations of generalized violence, violations of human rights, or natural or human-made disasters, and who have not crossed an internationally recognized state border.
**Recruitment** [7,9]	Compulsory, forced, or voluntary conscription or enlistment of children into any kind of armed force or armed groups.
**Refugee** [20]	A person, who is legally recognized as having a “well-founded fear of persecution for reasons of race, religion, nationality, membership of a particular social group or political opinions, is outside the country of his nationality and is unable or, owing to such fear, is unwilling to avail himself of the protection of that country.”
**War Crimes** [9,21]	War crimes are serious violations of international humanitarian law, giving rise to individual criminal responsibility. They include, but are not limited to, grave breaches of the Geneva Conventions. Examples include torture or inhuman treatment; killing or wounding a combatant who has surrendered or is otherwise out of combat; rape; making medical or religious personnel, medical units or medical transports the object of attack when they are not military objectives.

**Table 2:** The six grave violations against children during armed conflict [9,19].

Grave Violation	Description
**1. Killing and maiming of children**	The deliberate or incidental harm to children through violence, leaving them dead, severely injured, or permanently disabled. This violation often results from direct attacks, indiscriminate violence, or the use of explosive weapons in populated areas.
**2. Recruitment or use of children as soldiers**	Forcing or coercing children into roles as soldiers, spies, messengers, or human shields in armed forces or groups. This practice robs children of their innocence and places them in life-threatening situations.
**3. Sexual violence against children**	Encompasses rape, forced marriage, trafficking, and other forms of sexual abuse that target children, often used as a weapon of war to terrorize communities and destroy lives.
**4. Abduction of children**	The seizure of children from their homes, schools, or communities for exploitation, indoctrination, or forced participation in armed groups. Abductions traumatize children and violate their fundamental rights to safety and family.
**5. Attacks against schools or hospitals**	The deliberate targeting or use of educational and medical facilities, depriving children of critical access to learning and healthcare, and destroying spaces meant to protect and nurture them.
**6. Denial of humanitarian access for children**	Blocking or impeding humanitarian aid, preventing children in conflict zones from receiving essential food, water, medicine, and other life-saving resources, exacerbating suffering and mortality.

The terminology surrounding children’s protection in armed conflict is extensive and complex, much of which rests on the concepts of fundamental humanitarian principles (Table 3). Terms involving conflicts and children are often misused, confused, or even misconstrued in publications and public discourse, which can obscure the fundamental truth that children have inviolable rights that must be upheld. For example, terms such as *genocide* and *ethnic cleansing* carry distinct legal definitions under international law, yet they may be used interchangeably or inaccurately in media and advocacy, confusing discourse and preventing recognition of and accountability for atrocities, regardless of how they are classified. In addition, organizations may grapple with internal tensions between core humanitarian principles such as neutrality, impartiality, and *témoignage,* which may complicate decisions on how and when to respond to violations involving children while potentially jeopardizing access to vulnerable populations. By ensuring consistency and accuracy in terminology and concepts, we can create a shared language that fosters clearer communication, strengthens advocacy efforts, and reinforces the recognition of fundamental rights and of children as a protected group.

**Table 3:** Selected humanitarian principles relevant to children in armed conflict settings [22,23].

Humanity	The belief that human suffering must be addressed wherever it is found, with particular attention to the most vulnerable.
**Impartiality**	The belief that humanitarian care should be provided to all equally without discrimination. Decisions must be made on a “needs only” basis and must not be influenced by personal considerations or feelings.
**Non-discrimination**	The belief that humanitarian aid must help people regardless of their religious beliefs, the colour of their skin, their political convictions, their sexual orientation or gender identity, where they come from, or whether they are rich or poor.
**Proportionality**	The belief that whether treating the wounded or distributing food, aid provision must ensure that those in greatest need receive assistance first.
**Neutrality**	The belief that humanitarian aid must not favor any side in an armed conflict or other dispute.
**Independence**	The autonomy of humanitarian actors to not be subject to control, subordination, or influence by political, economic, military, or other objectives.

## Unique vulnerability of children during armed conflicts

*“Among the rubble, I searched for my family. I wanted to know if anyone had survived. But we couldn’t find them: not my father, not my mother, not anyone.*” Sahid, 9 years old, Gaza [24]

Children living in conflict zones are uniquely exposed to human rights violations, impacts on their physical and mental health, and erosion and destruction of their community’s social cohesion. [2–4,20] Armed groups frequently employ tactics that disproportionately harm children, including collective violence and the specific targeting of children and families. [2–4,20] Children may be targeted when traveling to and from school, [8] or they may be targeted if they reside in homes belonging to individuals of opposing political forces (e.g., Syria under the Al Assad regime or in Myanmar with the Rohingya in Rakhine state). [25,26] During conflict, families may be forced to flee their homes, seeking safety either within national borders as internally displaced persons or across international boundaries as refugees or asylum seekers. [2–4,20] Such displacements are rarely planned, often undertaken under extreme duress, and accompanied by significant hardship. [2–4,20]

As conflicts persist, the immediate chaos of acute instability gives way to prolonged and systemic disruptions, deepening the socio-economic impacts on children and their families. [2,4,13] Conflict disrupts food systems by destroying agricultural infrastructure, blocking supply chains, and limiting or impeding humanitarian aid; this can lead to food insecurity and even famine, which disproportionately affects children via malnutrition and increased mortality rates (e.g., in Sudan, Yemen, Palestine, and Ethiopia). [27–31] Schools and universities may be closed, destroyed, repurposed, targeted, or used as bases by combating parties, all of which sever children’s access to education and futures (e.g., in Sudan, more than 90% of the country’s 19 million school age children are unable to access formal education). [8,32,33] Conflict-related disruptions in healthcare services lead to lapses in vaccination rates, gaps in early treatment of common causes of morbidity and mortality in children under 5-years-old, and poor access to specialist and referral care. [20,34–36] Conflict deprives children of access to clean drinking water by damaging vital infrastructure, limiting access to safe water sources and proper sanitation, leaving families vulnerable to dehydration, and causing disease outbreaks (e.g., in Gaza, more than 2 million Palestinians lack access to water). [2,29] With the chronic breakdown of infrastructure and services, the cohesive fabric that binds communities together is frayed, and trust in systems erodes.

Additionally, children displaced by conflict or separated from their caretakers face uniquely heightened dangers. They are at risk for exploitation, abuse, trafficking, enslavement, and recruitment by armed groups. [4,7,10] Once separated from their caretakers, they are particularly vulnerable to illness, injury, and death as they navigate unfamiliar and often hostile environments. (e.g., in South Sudan, more than 65,000 children have been separated from their caregivers). [7,10,24] Children may also be abducted, detained without due process, or used as hostages or tools for negotiation by armed actors (e.g., as in Syria and Iraq in 2014, when the Islamic State abducted Yazidi children; in Nigeria in 2014, when Boko Haram abducted schoolgirls; Israel in 2023, when Hamas abducted Israeli children from their homes; and in Palestine, where children have been arrested and detained by the Israeli military). [37–40] Children may be separated from their families in order to erode cultural identity and promote indoctrination (e.g., in Ukraine in 2022, thousands of Ukrainian children were reportedly abducted by Russian forces). [41] Children, especially girls, may face sexual and gender-based violence (SGBV), child marriage, or forced labor, adding to the layers of harm inflicted by displacement (e.g., in eastern DRC, where children accounted for up to 45% of the nearly 10,000 documented sexual violence cases in January and February 2025). [7,42,43] Orphaned and unaccompanied children may be the lone survivors of armed conflict, as expressed by the devastating label *WCNSF* or *“wounded child, no surviving family”* currently used by medical professionals in Gaza. [44] The mental health toll from the effects of armed conflict on children is profound, with many children suffering from depression, bereavement and grief, anxiety, acute and chronic stress, trauma responses, and other conditions linked to the violence they have witnessed or endured.

Further details of the consequences of armed conflict on children are described in detail elsewhere, [2,4] and collectively, they underscore the urgency of concerted action to recognize children’s distinct claims for special protections in armed conflict settings.

## Defining témoignage

*“WHOEVER STAYS UNTIL THE END, WILL TELL THE STORY WE DID WHAT WE COULD. REMEMBER US.” -* Words written on a white board at Al Awda Hospital, Gaza, on October 20th, 2023, by Dr. Mahmoud Abu Nujaila, who was killed in an airstrike on November 21st, 2023. [45]

*Témoignage*, a French term rooted in humanitarianism, means to bear witness by observing and speaking out about injustice [23]; more simply, it means *to see and then to say what one saw*. *Témoignage* in conflict settings is not about taking sides, assigning blame, or proposing solutions; rather, it is the powerful act of recounting events, speaking on what one personally witnessed. [46] *Témoignage* can serve secondary purposes beyond firsthand witnessing and sharing of events and experiences. [46] It can contribute to the identification of perpetrators and serve as a call for accountability, both political and legal, when linked to one’s own direct experiences. [46] For example, when speaking out about violations in children’s rights, *témoignage* may trigger reporting mechanisms from actors in specialized monitoring, reporting, and legal spheres (e.g., UN-led Monitoring and Reporting Mechanism for the Six Grave Violations). [19,27,46–48] It may also prompt advocacy for child-focused legal accountability through international judicial systems (e.g., the International Criminal Court and the International Court of Justice). [48] Furthermore, the act of witnessing others’ *témoignage* itself becomes an extension of bearing witness, transforming individual experiences into collective awareness and opportunities for action.

As pediatricians, one of our most fundamental obligations is to uphold the principle of *témoignage*. This responsibility extends across the spectrum of our work, from advocating for policy changes that address harm to children to speaking out to colleagues, the public, and the media when we witness harm to children. [49,50] This advocacy, the essence of pediatrics, becomes particularly crucial when children are impacted by armed conflicts, made invisible in data and denied a voice in times of crisis. [27,51–54] Pediatricians individually can practice *témoignage* by drawing attention to injustices and inspiring those with power to enact meaningful change. [19,54] More broadly, pediatric associations and institutions can collaborate with experts in human and child rights, integrating pediatric perspectives into broader advocacy and addressing the violations and atrocities children face. [54]

Far more than reporting facts, *témoignage* is a *cri du cœur* – an impassioned moral outcry from the heart--that demands children in conflict settings be considered as a special population worthy of heightened attention, action, and justice. *Témoignage*, therefore, includes embedded protections for children upon which advocates can capitalize to maximize the effectiveness of humanitarian responses and influence systemic policy shifts. *Témoignage* calls on pediatricians to collectively shoulder the emotional and ethical weight of the atrocities we witness, in purposeful solidarity, carrying forward the stories of those affected to raise awareness, advocacy, and change. Through *témoignage*, the collective voice of many pediatricians holds tremendous power to contribute meaningfully to the global fight for justice and the protection of children in conflict settings.

## Ethical témoignage

*“‘They forget about us,’ one exhausted doctor said. ‘It’s a forgotten war.’”* - unnamed Sudanese physician, quoted on October 27, 2024. NPR [55]

Ethical *témoignage* demands that the voices of those in conflict settings be communicated faithfully, without distortion, embellishment, misrepresentation, or the insertion of assumptions. Particularly for children, *témoignage* carrying their voices to the wider world must protect the integrity of what they can and cannot express, knowing that their stories are *“shuffled, stuttered, always shattered beyond the repair of a narrative order*” [56]. Yet, despite the ethical imperative and even the best of intentions, it is easy to slip into unethical practices, particularly when entrenched power dynamics go unacknowledged, when certain voices remain invisible in mainstream media, and when stories are told without full, informed consent from those most affected.

The control of the narrative during conflicts often lies in the hands of powerful actors, shaping not only the response but also the perception of what is happening on the ground. [57,58] The complicated colonial legacies of systemic racism marginalize the lives and suffering of people predominantly in the “Global South,” where affected populations are framed as helpless and in need of rescuing from the “Global North.” [57] As author Arundhati Roy once said, “*There’s really no such thing as the ‘voiceless’. There are only the “deliberately silenced, or the preferably unheard*.” [58] In this context, humanitarian agencies often speak on behalf of affected populations and local healthcare workers instead of amplifying their voices, which obscures ownership of narratives, reinforces power imbalances, and misrepresents lived experiences. *Témoignage* is about speaking *with* affected populations, not *for* them, and pediatricians should leverage their unique positions, privilege, power, and credibility to prioritize the dignity, agency, and centrality of children and families enduring the conflict.

*Témoignage* on behalf of children also demands bearing witness to *all* conflicts, not just those amplified by media or political interests. Current crises in Sudan, South Sudan, Yemen, Ethiopia, Myanmar, Haiti, and DRC, among others, are underrepresented in the media and public discourse. [26,30,31,53,55,59–61] Affected populations may be entirely absent from the media or political dialogue, suggesting that their lives do not hold enough value to bear mention. This reflects a global system in which the visibility of suffering is dictated by geopolitical convenience, racial hierarchies, and the marketability of victims. [57,62,63] Bearing witness to the injustice and human suffering in these under-reported conflicts means acknowledging the inequities that shape international aid, advocacy efforts, and honoring the universal rights and dignity of all those affected.

In addition to amplifying unheard or silenced voices, ethical *témoignage* requires obtaining consent, a cornerstone of respecting the privacy and autonomy of those who own these stories. [52,64] Clear communication about how testimony will be used and ensuring individuals understand their right to not share stories helps prevent exploitation and further harm, especially for those already experiencing trauma. [52,64] Regarding testimony through photographs and other media, images of the suffering of children should never exploit their suffering, but rather, bring their experiences to the attention of the rest of the world with an intent for ethical response. [23,64] Additionally, safeguarding of identities is critical to protecting individuals from retaliation, stigma, or additional harm, particularly in conflict and military occupation zones. [52,64] Measures such as anonymizing data, omitting identifiable details, and carefully framing narratives are essential to maintaining safety and trust. [52,64] Together, these principles ensure that *témoignage* not only amplifies voices but also upholds the humanitarian commitment to protect and empower those most vulnerable.

## Témoignage as a tool of protection and recognition of the unique status of the child

*“We write, we scream, we document. But who reads? Who cares? Is the goal for us to disappear? To go silent? To die slowly?” -* Rita Baroud, Palestinian journalist and correspondent [65]

Some statements regarding children in conflict settings should never have to be said, such as that no child should be tortured, raped, or killed. Yet, time and again, violations of rights befall children in front of us all - at the time of writing, atrocities such as those discussed above have been reported in many active armed conflicts, including Burkina Faso, Cameroon, Central Africa Republic, Chad, Colombia, DRC, Ethiopia, Haiti, Israel, Lebanon, Mali, Myanmar, Niger, Palestine, Sudan, Syria, Ukraine, and Yemen [2,19,26,28,29,31,37,39,44,55,66,67]. It is our duty as pediatricians to assert that all children require an additional level of protection above other civilians (including for unaccompanied minors and children associated with armed forces and armed groups), and visibility on the world stage regardless of their geographical location. This level of protection embedded in *témoignage* can occur via the following means:

1 **Advocating for the “Child Primacy Norm”**

*“We ought to assess everything we do from the position of trying to ensure the welfare of children. That’s it, the elevator pitch to save the world.”* - Adam Benforado, Professor and author [68]

The principle of *témoignage* compels us to observe and speak out against injustices, especially when they endanger the most vulnerable. This principle underpins the advocacy for the *“child primacy norm,”* which emphasizes placing children’s well-being at the forefront of all policy decisions. [68–70] Other scholars and advocates who have explored the UNCRC and how it applies in various contexts have articulated that children’s rights and their best interests must be taken seriously and considered in all matters that affect their welfare. [69,70] When an activity poses a significant risk to children, the default response should be to halt, gather information, explore alternatives, and address the threat, with the burden of proof falling on those creating the potential risk. [2,68,70] In conflict zones, the child primacy norm demands that those engaged in warfare ensure their actions do not endanger children, such as avoiding targeting schools, places of worship, or civilian areas. [2,68] As pediatricians and witnesses to the devastating impact of these actions, we have an ethical responsibility to advocate for a transformation in sociopolitical norms—a shared framework that places children’s rights, safety, and well-being at the center of both conflict and peacetime policies. By doing so, we ensure that children’s needs and protections remain integral to societal and governmental decisions across the globe.

2 **Advocating for the special protection of children in armed conflict settings**

*“I’ve spoken to mothers who sleep with one hand wrapped around their remaining children, and another around photos of the ones they buried under rubble… And I’ve spoken to children who think war is normal, because it’s all they’ve ever known.”* – Nour El Assy, Palestinian poet and writer [71]

The principle of *témoignage* calls for a global commitment to protecting children in conflict settings through specialized, not merely equal, safeguards that go beyond those afforded to the general civilian population. Unlike equality, which assumes identical treatment for all, effective protection of children requires tailored approaches that address their distinct vulnerabilities based on age and developmental stage. Prioritizing their safety and well-being means implementing child-specific measures, such as designated safe zones in conflict, uninterrupted access to education, trauma-informed approaches to their care, and accountability mechanisms for crimes against children. [8,47] Additionally, recognizing children as a distinct and politically acknowledged group reinforces the shared responsibility of the governments, international organizations, and civil society to uphold their rights and ensure their representation in decisions affecting their survival and future. Despite the clear need for these distinct protections, historical and political obstacles have hindered their full implementation. Challenges include resistance to recognizing children as independent rights holders in conflict settings, limitations in legal accountability mechanisms, and competing priorities within international humanitarian responses. [3] Notably, while the UNCRC is the most widely ratified human rights treaty in history, the United States remains the only UN member state that has not ratified it. [72] Addressing these barriers is crucial to advancing a framework where children’s rights in armed conflict are not just acknowledged but also actively enforced, protected, and promoted. [3,70]

3 **Advocating for the protection of healthcare workers and children’s medical care**

*“In war, I said, we talk of the fall of cities. The Fall of Mosul. The Fall of Saigon. I asked when it became normalized to speak of the Fall of Hospitals. The Fall of Al-Shifa. The Fall of Al-Aqsa Hospital.”* - Dr. Seema Jilani, pediatrician, humanitarian aid worker, and author [73]

Ensuring a child’s access to medical care is a cornerstone of public health and a reflection of a functioning society. [74] Functional pediatric care systems, including maternal and newborn health services, vaccination programs, and pediatric emergency response, are a critical component of safeguarding broader civilian populations and serve as vital pillars for entire communities. [2,13] Healthy, thriving children contribute to the well-being of their communities, which can be particularly important to the community’s resilience and recovery during crises. [74] When health systems collapse or are deliberately attacked in armed conflict, not only are children disproportionately impacted by disruptions to medical care, but the ripple effects upon a population can last for generations. [2,13,30]

Bearing witness goes beyond advocating for individual children, however, and involves amplifying the plight of those providing their care under perilous conditions. Despite protections outlined in international humanitarian law, hospitals and healthcare workers are increasingly becoming targets of war, with thousands of incidents of violence or obstruction reported annually. [35,75,76] In 2024, there were 1,602 documented incidents of violence against healthcare workers or healthcare systems in armed conflict, spanning 15 countries or territories, resulting in the deaths of 933 healthcare workers. [77] These attacks—including bombings of facilities, hijackings of ambulances, and assaults on personnel—devastate health systems and restrict children’s access to care. [35,77] Impunity to such attacks has consequences elsewhere; these numbers have continued to rise each year, with such violence becoming increasingly normalized and disproportionate in current conflict zones. [76] Healthcare workers who remain in conflict zones often do so under extraordinary hardship, contending with inadequate supplies, power shortages, constant exposure to danger, and their own personal trauma, grief, and loss. [35,45] Consequently, many endure profound physical and emotional trauma, while others are forced to flee, further destabilizing already fragile healthcare systems and leaving children without appropriate and specialized care. [35] Bearing witness to these realities involves not only documenting these acts of violence but also advocating that a child’s right to access healthcare and the protection of health workers are never compromised.

4 **Confronting the dehumanization of children**

*“The idea that some lives matter less is the root of all that is wrong with the world.”*

*—*Dr. Paul Farmer, physician, medical anthropologist, and co-founder of Partners in Health [78]

Dehumanizing narratives often strip children in conflict of their vulnerability and humanity, labeling them as “*human shields,” “child soldiers,” “terrorist supporters,”* or “*future terrorists.”* This harmful language obscures the reality that children lack the agency to control their circumstances and deserve special protection. Terms such as *Children and Girls Associated with Armed Forces or Groups* have been developed to counteract this rhetoric by acknowledging children’s unique vulnerabilities and absolving them of any blame for their association with armed groups. [7] Through *témoignage*, pediatricians and humanitarian stakeholders must challenge these damaging narratives and advocate for language that humanizes children, upholds their blameless status, and respects their inherent dignity. [7] Moreover, research into “re-humanization” through discourse remains a neglected area, particularly concerning children associated with armed forces and groups. [79] Creating safe platforms for children to share their stories and voice their concerns is equally critical, fostering empathy and understanding while advancing efforts to restore their humanity and protect their rights. [79]

5 **Ensuring the Visibility of Children Through Data-Driven Témoignage**

*“As we left our neighbourhood, we saw people who had been shot laying on the ground, covered in blood.… There were so many people in the same situation as us. Seeing all these people with bags and other belongings fleeing their neighborhoods was quite strange.” -* Jean, 9 years old, Haiti [67]

Although *témoignage* is defined by personal testimony and narrative, contextualizing the stories in data adds depth and nuance, lends credibility, provides a sense of scale, and improves children’s visibility in conflict. While humanitarian actors collect child public health data in all but the most extreme contexts, when every child is not counted, there are important gaps in data practices that perpetuate invisibility and voicelessnes. [13,80] Healthworkers caring for children from conflict-affected populations are well-placed to address these gaps and can play a critical role in improving the quality, relevance, and timeliness of child public health data collection. Data collected may demonstrate patterns of harm (such as changes in age-specific mortality, causes of mortality, prevalence of malnutrition and disability, and impacts on child development) and also provide opportunities to improve humanitarian responses for children while monitoring the safety and effectiveness of interventions.

6 **Reconsidering neutrality**

*“We must always take sides. Neutrality helps the oppressor, never the victim. Silence encourages the tormentor, never the tormented”*—Elie Wiesel, Holocaust survivor, author, and activist [81]

Neutrality in humanitarianism - the belief that aid must not favor any side in an armed conflict or other dispute (Table 3) [22] - remains a debated principle in humanitarian circles. [63,82] While neutrality is important for impartiality (i.e., the provision of aid solely based on need and without discrimination) and maintaining access to all parties in a conflict, [22] strict adherence may silence humanitarian actors in the face of atrocities. [82] This may prevent them from advocating for vulnerable populations like children and risk moral compromise and complicity in human rights violations. [22,63,82] Additionally, *“humanitarian whitewashing,*” or the concept that countries may use participation in humanitarian efforts to hide their own human rights abuses, further contributes to complicity in an otherwise ‘“neutral’” aid response. [83]

Children, because of their special status, require more than aid provision within a strict framework of neutrality. They require advocacy for the truth, particularly in the face of violence and systemic violations of their rights. Such truth-telling is inherently in line with the practice of pediatrics, and it is truth - not neutrality - that empowers action to disrupt cycles of violence, expose perpetrators, and ensure that the rights of children are not overlooked during conflict. However, a commitment to neutrality and impartiality is critical in maintaining access to children, no matter on which side of a conflict they happen to be born. In practice, balancing *témoignage* for children against neutrality creates tension in humanitarian responses, presenting a difficult but necessary challenge for humanitarian actors to navigate the fine line between transparency and maintaining access to populations while not shielding perpetrators from justice, obstructing efforts to end violations, or obfuscating the truth when the time for accountability arises.

Thus, neutrality must be redefined as a dynamic, context-driven, and integral part of humanitarian response that preserves access to children while preventing complicity in harm, injustice, and the passive or active support of perpetrators. By embracing this nuanced approach, pediatricians can acknowledge the ethical contradictions inherent in humanitarian work while upholding their duty to provide equitable care, challenge the inequities that continue to shape aid responses, and advocate for all children in any conflict.

### Risks and challenges of *Témoignage*

*“Only one thing is more frightening than speaking your truth. And that is not speaking” -* Audre Lorde, writer, poet, and civil rights activist [84]

Speaking out against injustices to children in the context of armed conflicts carries significant risks and challenges. Healthcare workers on the ground who practice *témoignage* risk retaliation and hazards to themselves and to their communities, families, or lives for daring to bear witness to atrocities. [45,85] They risk not only loss of livelihood, which can devastate entire households, but also the threat of forced displacement, detention, abduction, physical violence, or death. [75,76] For many, the risks are compounded by limited access to legal protections or mechanisms for redress, leaving them vulnerable to unchecked abuses. [86] These realities may compel individuals and organizations to adopt cautious stances, prioritizing self-preservation over advocacy, particularly in settings where authoritarian regimes or powerful entities may control the public narrative and punish dissent. This dynamic underscores the immense courage of those on the frontlines and highlights the unequal burden they bear in speaking out for children when atrocities occur.

For pediatricians who raise their voices from outside of conflict zones, *témoignage* may bring targeted retaliation to their careers and reputations. [73,87,88] They may face censorship of publications, cancellation of speaking events, expulsion from professional societies, and disciplinary measures by their institutions, all of which infringe upon academic freedoms, compromise intellectual rigor, and actively silence dissent. [73,87–89] Discussions on social media networks and other online platforms - where disagreements may arise over the objectives of a conflict, the military means employed, or the role of governments, actors, and humanitarian organizations [90] - may amplify tensions, making it increasingly difficult for pediatricians to navigate the contentious terrain of advocacy without encountering criticism or retribution. These consequences may deter physicians from engaging in critical discourse on the impact of armed conflict on children.

As a specific example, the politicization of advocacy for children in armed conflict settings is evident in discussions surrounding the current Israel-Palestine conflict. Members of this author group have encountered reprimands, censures, cancellations, and threats for speaking, writing, or sharing publicly their experiences with or perspectives on the current plight of children in Palestine. [73,85,89] Other healthcare professionals have described backlash after advocating for children killed or taken hostage during the attack on Israelis on October 7^th^, 2023. [91–94] While pediatricians have long spoken out against the suffering of children in war zones, similar advocacy concerning children in other current conflicts has not provoked comparable responses. [25,31,38,39,41,46,53,54,66,95,96] The contentiousness around this conflict—and the ongoing lack of attention to others—raises a critical existential query: *Whose suffering is deemed justifiable?*

It should also be noted that bearing witness to the suffering of children is undeniably distressing. Humanitarian pediatricians may experience moral injury when they are unable to speak out against atrocities perpetrated on children, when they have exercised their privilege to exit conflict zones but left behind local colleagues or patients whose stories they share, or in cases when they have spoken out but then experience the painful gap between the suffering they witness and the actions they are able to take against it. [62] *Témoignage,* therefore, requires vulnerability, not only from those who share their stories but also from those who hear them. It demands the courage to speak but also the courage to listen, to sit with discomfort, to confront painful truths, and to remain present even when realities are difficult or inconvenient to hear. The burden of witnessing does not rest solely with the storyteller. Those who receive these stories carry a mirrored burden – the responsibility to reflect, respond, and act. This process can be emotionally taxing, particularly for professionals who may experience vicarious trauma as a result. [97] If pediatricians can learn to carry the weight of shared stories without being crushed by them, the vulnerability associated with bearing witness also opens the door to a deeper form of solidarity. The shared nature of this responsibility echoes Dr. Paul Farmer’s concept of *accompaniment*, which entails that, *“the moral responsibility of the healer [is to] step inside [another’s] experiences and accompany them through the worst moments with empathy and expertise, compassion and care, for as long as it takes.”* [98]

Some may worry that these stories are not theirs to tell. Others may fear that sharing such narratives reduce people to their suffering alone, defining them solely by the trauma endured rather than honoring their full dignity and humanity. Still others may hesitate out of concern that their storytelling could offend, misrepresent, or spark controversy. And so, out of caution or fear, they choose silence over speaking out. Yet, we must acknowledge the risks of remaining silent. Failing to speak out due to fear creates a void that can be quickly filled with misinformation, propaganda, or harmful narratives, enabling perpetrators to continue committing atrocities unchecked. Silence, far from neutrality, is an act of abandonment and complicity which exonerates the aggressor. Silence signals implicit consent to systems of oppression and violence, undermining the moral responsibility of pediatricians to bear witness to the suffering of children. To speak out, to name atrocities, and to draw attention to their impact, is to reject complicity in silence. While this advocacy may entail personal, institutional or organizational risk, the cost of inaction—to children enduring profound suffering and to the moral integrity of the global community—is far greater.

## Solidarity in témoignage

*“I go forth alone and stand as ten thousand.”* – Maya Angelou, memoirist, poet and civil rights activist [99]

To bear witness on behalf of children in conflict is to enter a longstanding tradition of advocacy—one grounded in solidarity and shaped by the voices of those who have led the way, both past and present. All those who practice *témoignage* for children in conflict settings can rely on the profound understanding that they do not speak alone. Each act of advocacy joins a global chorus of pediatricians, humanitarians, caregivers, and affected children who have long demanded justice, safety, and dignity for children. This collective voice reinforces individual efforts, offering both solidarity and a powerful reminder that the struggle for children’s rights is shared, deeply rooted, and enduring. One of the greatest protections for *témoignage* is this very solidarity—a unified front that not only amplifies truth but also fortifies those who bear witness against isolation, retaliation, or despair. In this way, *témoignage* becomes more than testimony; it becomes a catalyst for collective action, accountability, and meaningful change—even in the face of profound adversity.

## Témoignage in action

*“‘Tell them: we are tired. We are without homes, on the streets, and our loved ones are gone and we are all stories.’”-* Dr. Tanya Haj-Hassan, pediatric intensivist and humanitarian aid worker, recounting the words of a Palestinian nurse, at the United Nations [85]

Ultimately, our role as pediatricians extends beyond treating the physical and emotional wounds inflicted by armed conflict; it calls on us to envision a better future and to advocate for and build a world that reduces suffering. In a world increasingly shaped by the *“globalization of indifference,”* [100] the impulse is to look away from the suffering of children. Faced with the endless, real-time images of violence against children in news and social media, our humanity is tested—we risk desensitization, apathy, and paralysis. Some look away, refusing to see; others see clearly, but choose the comfort of inaction. For those with the privilege to disengage, avoiding the horrors of war upon children can feel like self-preservation—but it comes at the cost of our shared humanity.

**Fig 1:**
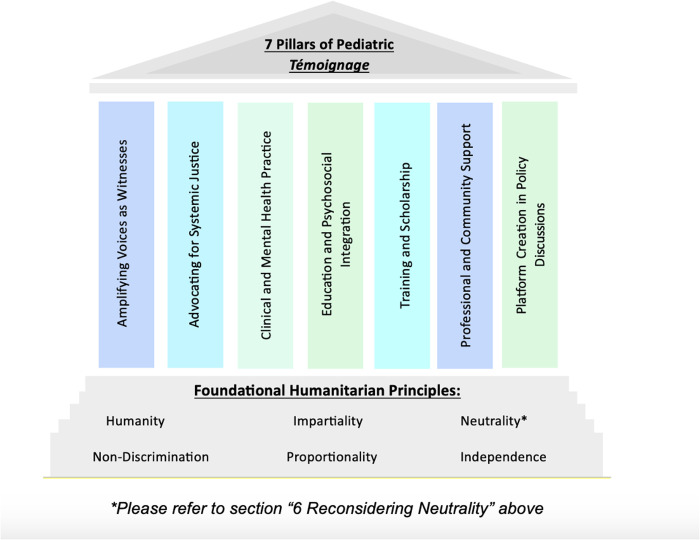
Seven Pillars of Pediatric *Témoignage* for Children in Armed Conflict Settings.

To use the protection embedded in *témoignage* to elevate the special claims and rights of children affected by conflict, we propose seven pillars of pediatric *témoignage* for children in armed conflict settings (Fig 1), which are based upon the foundational humanitarian principles outlined in Table 3. [22,23] In Table 4, we provide actionable recommendations to implement these pillars in pediatric practice, whether working within conflict settings or from a privileged place of safety.

**Table 4:** Examples of *Témoignage* for Children in Armed Conflict Settings.

Pillar	Examples of *Témoignage* from Pediatricians or Pediatric Societies
*Témoignage* through Amplifying Voices as Witnesses	• Amplify the voices and stories directly from children and colleagues in conflict settings, ensuring their experiences are heard in professional, policy, and public forums and shape advocacy efforts. • Use organizational platforms at conferences and professional gatherings to amplify the voices of children, families, and caregivers affected by armed conflict, sharing their stories in policy and public forums. • Facilitate platforms for children and families to share their lived experiences with policy makers, emphasizing their role in shaping effective interventions and policies. • Model and promote the use of accurate and respectful terms in social media and professional discussions, promoting a narrative that respects the dignity and rights of children in conflict. • Speak up in professional dialogues to redirect contentious discussions toward *témoignage*, appropriate terminology, and the universal rights of children. • Share resources on immigrant and refugee populations, highlighting conflicts affecting them to educate colleagues and policymakers. • Vocally support experts and organizations that act against breaches of children’s rights and amplify their work locally, nationally, and internationally. • Partner with media organizations to document and share stories of children and colleagues in conflict settings, ensuring accurate and ethical representation of their experiences and needs.
*Témoignage* through Advocating for Systemic Justice	• Advocate for legal representation for children in asylum hearings—ensuring their needs and voices are centered in the judicial process—and for both children and their parents to have access to legal representation when detained or arrested in conflict settings. • Call for funding and policies that provide interpretation services, ensuring equitable access to mental health care for non-English-speaking children. • Challenge harmful stereotypes and discriminatory narratives about refugee and migrant children, using *témoignage* to redirect discussions toward empathy and justice. • Push for sustainable, community-led solutions to address gaps in services for children affected by conflict, advocating for the inclusion of local stakeholders in decision-making processes.
*Témoignage* through Clinical and Mental Health Practice	• Advocate for equitable access to healthcare and evidence-based care for CIAC, using your platform to call for systemic changes in service delivery. • Advocate for culturally sensitive and trauma-informed clinical care in practice locations; use your voice to advocate for improved protocols and training when these standards are unmet. • Advocate for use, creation, and open access of clinical guidelines that prioritize the emotional and psychological needs of children, siblings, and caregivers impacted by conflict. • Speak out against discriminatory practices and language within healthcare settings, ensuring that all children and families are treated with dignity. • Advocate for culturally sensitive psychoeducation for families managing conflict-related stress, including the impact of distressing media coverage. • Share insights from your clinical practice to call attention to the gaps in resources for children exposed to armed conflict, emphasizing the need for psychological first aid and long-term mental health and social support. • Partner with refugee resettlement organizations to amplify their calls for mental health services and rehabilitation resources, lending professional credibility to their efforts. • Use your platform to push for increased funding for clinical and psychosocial support programs, emphasizing the urgent mental health needs of displaced children and families. • Advocate for distance-support programs for colleagues working directly in conflict settings, based on the gaps and needs those health workers have identified.
*Témoignage* through Education and Psychosocial Integration	• Advocate for the integration of mental health and psychosocial support into school programs, ensuring the psychological needs of children affected by armed conflict are addressed long term. • Champion education programs (e.g., online, app- or radio-based) that keep children engaged during and after conflicts and displacement. • Advocate for accessibility to education during conflict and displacement for all children, particularly those with disabilities or from marginalized groups (e.g., children associated with armed forces, child-headed households). • Call for trauma-informed and grief-sensitive training for teachers and school staff, enabling them to support children recovering from conflict-related trauma and loss. • Volunteer with and advocate for funding for local organizations to amplify their efforts in providing educational support and resources to displaced children. • Collaborate with community organizations to design curricula and activities that reflect the lived experiences of conflict-affected children, addressing their specific psychosocial needs. • Amplify the work of grassroots educational initiatives supporting displaced children, advocating for additional professional and financial resources.
*Témoignage* through Training and Scholarship	• Advocate for pediatric-specific training for healthcare workers in conflict zones, focusing on trauma care, bereavement support, and ethical responsibilities in challenging environments. • Speak out in professional forums to advocate for research on the health and developmental impacts of armed conflict, prioritizing trauma- and grief-sensitive methodologies. • Use conferences and academic platforms to share insights from local stakeholders and amplify their contributions to understanding the needs of children in conflict. • Advocate for research partnerships with local organizations to ensure findings reflect community-specific needs and inform sustainable responses. • Advocate for the inclusion of local voices in research to ensure findings accurately reflect community needs. • Encourage the publication of research findings on the developmental and health impacts of armed conflict on children, ensuring their dissemination shapes policy and clinical practices.
*Témoignage* through Professional and Community Support	• Support the advocacy efforts of reputable and responsible humanitarian aid organizations and refugee agencies by amplifying their calls for resources and action. • Speak out about the need for evidence-based care and equitable access to health services for children who experienced conflict, highlighting the critical gaps in care that require immediate action. • Advocate for the meaningful involvement of local stakeholders in designing interventions, ensuring solutions align with community needs and priorities. • Partner with community leaders to amplify their calls for resources and policies that support children and families affected by armed conflict. • Use your professional credibility to validate the funding and resource requests made by refugee resettlement organizations, emphasizing their importance for displaced children’s recovery.
*Témoignage* through Platform Creation in Policy Discussions	• Advocate for and organize local or national policy briefings where testimonies from children, families, and caregivers in conflict settings are central to discussions on solutions for the breeches of children’s rights. • Advocate for federal funding of programs that prioritize storytelling and *témoignage* from conflict-affected populations, ensuring their narratives influence legislative priorities. • Promote the recognition of testimony-driven advocacy as a cornerstone of systemic reforms for children affected by conflict, emphasizing its role in policy innovation.

Our pillars and recommendations require thoughtful partnership with expert organizations and the creativity and courage to seek out opportunities to collaborate with, advocate for, and amplify the voices from groups directly engaging with children in these settings. We focus on amplifying the voices of children and their caregivers, ensuring their stories are shared in public, professional, and policy forums. We advocate for systemic justice, challenging discriminatory practices that hinder policies aimed at safeguarding children’s unique rights and access to education and healthcare. We recommend integrating trauma-informed practices and adequate psychological and emotional support into the societal frameworks for children. Additionally, we propose advocating for training, research, and collaborative initiatives that enhance the understanding of and response to the impact of armed conflict on children. While these recommendations are ambitious and far-reaching, we believe that any pediatric healthcare provider anywhere in the world can find relevant suggestions on which they can build their own further actions.

## Beyond témoignage

*“...the war unfolding in the Democratic Republic of Congo remains an afterthought. A bloody conflict is met with condemnations but no meaningful action. This stark contrast is not just neglect; it is selective justice…. Our people deserve justice. Our children deserve a future. And the world must finally decide if the values it claims to uphold apply to all of humanity or only a chosen few.”* - Dr. Denis Mukwege, physician in the Democratic Republic of Congo [53]

*Témoignage*, no matter how compelling, is not enough on its own to prevent atrocities against children. The world has repeated the phrase “never again” countless times, yet mass suffering of children continues, often met with little more than rhetoric or justification. Governments, leaders, and international bodies have drawn moral and political red lines, declaring certain actions intolerable and grounds for intervention, but repeatedly these lines have been crossed with impunity. *Témoignage* may lay bare the horrors of violence against children, but without decisive action from those with the power to intervene, these testimonies risk becoming nothing more than echoes in the void or stories in history books. Furthermore, they may serve as tragic documentation of complicity in harm to children – evidence that the world knew and still did nothing. Moral clarity around preserving the child primacy norm must be matched by political will, and outrage at violations to unique rights of children must translate into accountability and justice. Otherwise, we risk normalizing the very horrors we claim to condemn.

## Conclusion

*“‘We are being buried. Every minute we disappear, every minute we are being abducted. We are experiencing things that the minds cannot even comprehend. We die and cannot find anyone to bury us. I’m asking you to share my story, my whole story, with my name. I want the whole world to know I am a human being. In the end, I am not pen on paper. I am not anonymous. I am a human being created by God.’”*

*-* Dr. Tanya Haj-Hassan, recounting the words of Saeed, a Palestinian nurse, at the United Nations [85]

While each of us may take vastly different paths in our journey of *témoignage*—shaped by our unique experiences, resources, and perspectives—pediatricians all abide by the same foundational principles that define our profession to allow children the opportunity and safety to grow and thrive. By holding steadfast to this core belief, we can overcome divisions, amplify our collective voice under the protection of solidarity, and insist that all children are human, and that children maintain a special status in conflict settings, one that demands a heightened and unique level of protection. If we are to be successful, we must collectively and courageously bear witness - for those who are silenced and to those we fear – even if it is difficult, even if our voices shake. We hold tremendous power as advocates for children worldwide. We must not remain silent when we can speak.
