# Correction: Bearing Witness: *Témoignage* as a Tool for Child Advocacy during Armed Conflict

**DOI:** 10.1371/journal.pgph.0006029

**Published:** 2026-02-11

**Authors:** The PLOS Global Public Health Editors

## Notice of Republication

This article [1] was republished on February 6, 2026, to address an affiliation issue and update the competing interest disclosure. Please download this article again to view the correct version.
